# Supplementary material for: Comprehensive fitness landscape of SARS-CoV-2 Mpro reveals insights into viral resistance mechanisms
Source: eLife. 2022 Jun 20;11:e77433. doi: 10.7554/eLife.77433 (PMC9323007; doi:10.7554/eLife.77433)
Supplement: Figure 4—source data 2. [file elife-77433-fig4-data2.docx]

**Mutation Mpro Relative cat. rate Functional score PMID**

|  |  |  | TF | FRET | growth |  |
| --- | --- | --- | --- | --- | --- | --- |
| R4A | CoV | 0.75 | 1.0 | 0.92 | 0.48 |  |
| R4E | CoV | 0.01 | 0.23 | 0.02 | 0.08 | 16329994 |
| K5A | CoV | 0.7 | 0.96 | 1.0 | 0.99 | 16329994 |
| M6A | CoV | <0.01 | 0.64 | 0.07 | 0.2 | 16329994 |
| P9T | CoV-2 | 0.03 | 0.0 | -0.01 | 0.0 | 33208735 |
| H41A | CoV-2 | 0 | 0 | 0.02 | 0.01 | 34249864 |
| H41D | CoV-2 | 0 | 0.06 | -0.02 | -0.02 | 34249864 |
| H41E | CoV-2 | 0 | 0.0 | 0.01 | -0.03 | 34249864 |
| P108S | CoV-2 | 0.85 | 1.0 | 1.05 | 1.02 | Abe et al, 2021 |
| S123A | CoV | 1.0 | 1.0 | .94 | 0.82 | 18275836 |
| S123C | CoV | 1.0 | 1.0 | 1.03 | 1.0 | 18275836 |
| S139A | CoV | 0.96 | 1.0 | 1.02 | 0.93 | 17154528 |
| S144A | CoV | 0.53 | 1.0 | 1.02 | 0.55 | 17154528 |
| C145A | CoV | 0 | -0.02 | 0.02 | -0.02 | 34249864 |
| C145S | CoV-2 | 0 | 0.21 | 0.0 | .04 | 34249864 |
| S147A | CoV | 0.01 | 0.27 | 0.03 | 0.08 | 17154528 |
| E166A | CoV | 0.5 | 1.0 | 0.99 | 0.78 | 20371333 |
| E290A | CoV | 0 | 0.0 | 0.02 | -0.03 | 15554703 |
| R298A | CoV | 0.11 | 0.79 | 0.18 | 0.27 | 18275836 |
| R298K | CoV | 0.53 | 1.0 | 1.03 | 0.83 | 18275836 |
| R298L | CoV | 0.15 | 0.74 | 0.12 | 0.19 | 18275836 |
| Q299A | CoV | 0.02 | 0.84 | 0.13 | 0.24 | 18275836 |
| Q299E | CoV | 0.07 | 0.33 | 0.01 | 0.07 | 18275836 |
| Q299K | CoV | 0.07 | 0.2 | 0 | 0.05 | 18275836 |
| Q299N | CoV | 0.05 | 0.93 | 0.44 | 0.49 | 18275836 |
